# Supplementary material for: A scalable proteogenomic framework for dissecting phospho-signaling pathways in primary immune cells
Source: bioRxiv. 2025 Oct 8:2025.10.08.681012. Preprint. [Version 1] doi: 10.1101/2025.10.08.681012 (PMC12632528; doi:10.1101/2025.10.08.681012)
Supplement: Supplement 1 [file NIHPP2025.10.08.681012v1-supplement-1.pdf]

bioRxiv preprint doi: https://doi.org/10.1101/2025.10.08.681012; this version posted October 8, 2025. The copyright holder for this preprint (which was not certified by peer review) is the author/funder, who has granted bioRxiv a license to display the preprint in perpetuity. It is made available under aCC-BY 4.0 International license.

Supplementary Figure 1

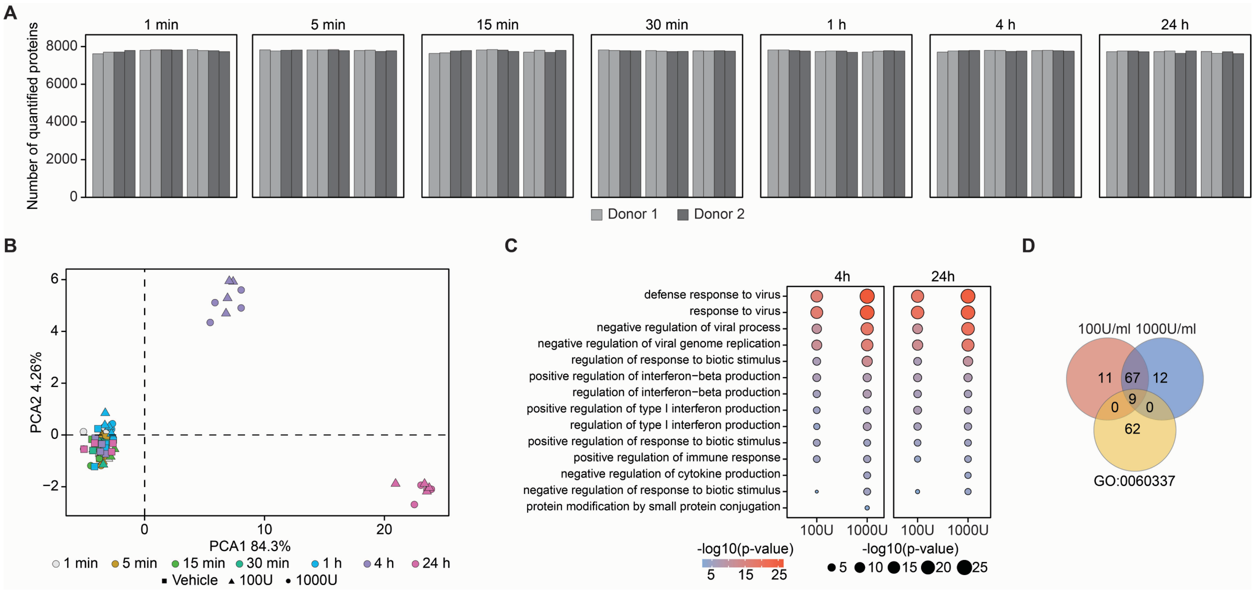

Supplementary Figure 1: Proteomic Overview of IFN-β–Treated Primary Human CD4 T Cells. (**A**) Number of quantified proteins in each proteomics sample. (**B**) Principal component analysis (PCA) based on significantly regulated proteins following IFN-β stimulation. (**C**) Gene Ontology (GO) enrichment analysis of significantly regulated proteins, highlighting pathways associated with interferon signaling. (**D**) Venn diagram showing shared and unique ISGs across the two treatment conditions, compared with proteins included in the Gene Ontology term GO:0060337.

bioRxiv preprint doi: https://doi.org/10.1101/2025.10.08.681012; this version posted October 8, 2025. The copyright holder for this preprint (which was not certified by peer review) is the author/funder, who has granted bioRxiv a license to display the preprint in perpetuity. It is made available under aCC-BY 4.0 International license.

Supplementary Figure 2

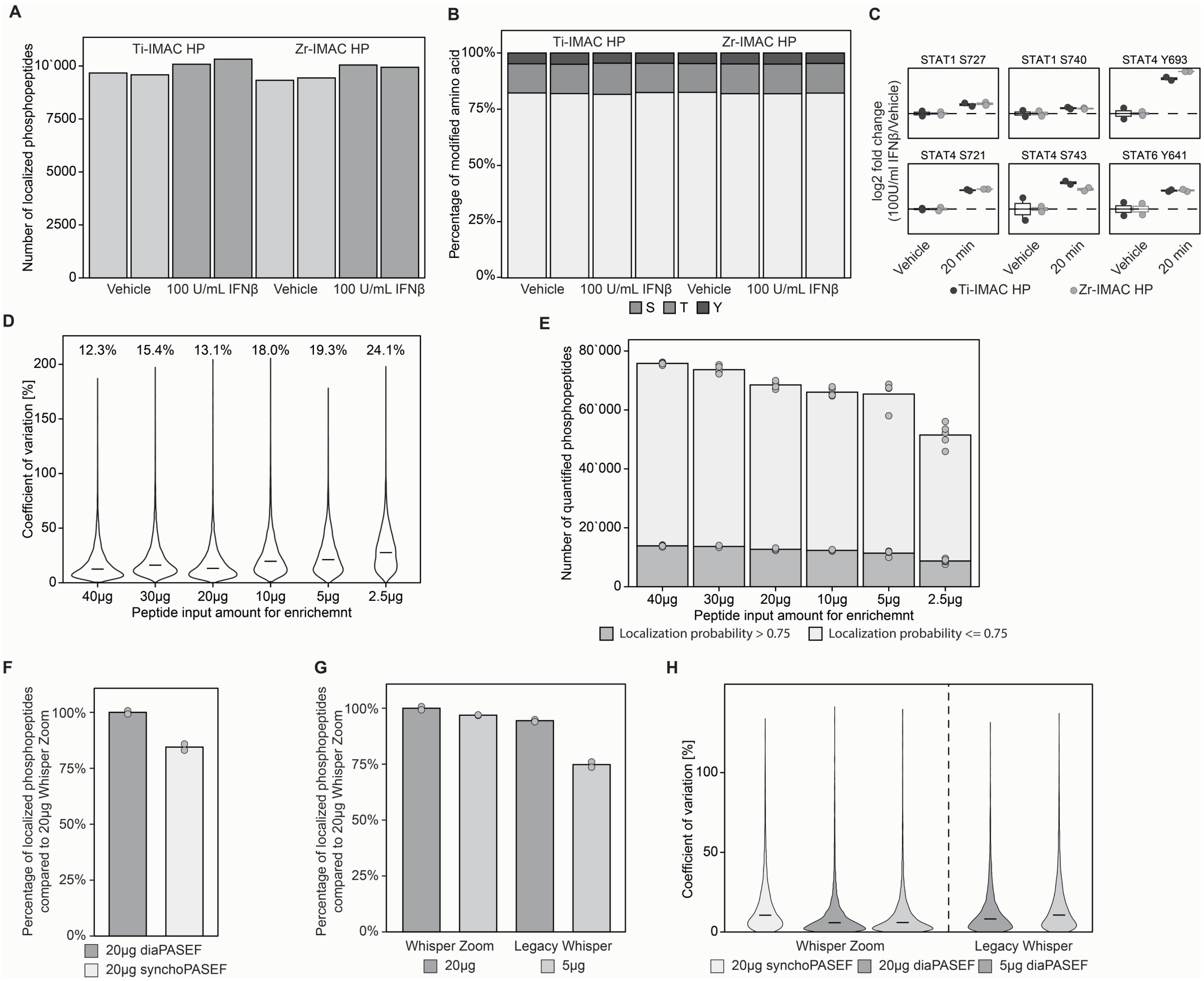

Supplementary Figure 2: Optimization of the simplePhos workflow for phosphoproteomics in primary immune cells. (**A**) Comparison of the number of class 1 phosphopeptides enriched using Ti-IMAC HP versus Zr-IMAC HP magnetic beads using CD4 T cells. (**B**) Bar plot showing the distribution of class I phosphopeptides by residue type, indicating the percentage of phosphorylation events occurring on serine, threonine, and tyrosine residues. (**C**) Box plots displaying differential phosphorylation of canonical STAT protein sites following interferon-β treatment, using enrichment with Ti-IMAC HP or Zr-IMAC HP beads. (**D**) Coefficient of variation (CV) of phosphopeptide quantification across biological replicates from a peptide input dilution series. (**E**) Quantification of class 1 and non-localized phosphosites across different peptide input levels to evaluate sensitivity and yield of confidently localized sites. (**F**) Number of localized phosphopeptides identified using either diaPASEF or synchro-PASEF acquisition modes on the timsTOF Pro2 platform, using 20 µg peptide input for the simplePhos enrichment, and the EvoSep Whisper Zoom gradient. (**G**) Impact of EvoSep chromatographic separation methods on phosphopeptide quantification using different peptide inputs. (**H**) CV of phosphopeptide quantification across replicates comparing multiple variables: Acquisition strategy (synchro-PASEF vs diaPASEF), EvoSep chromatography separation method (“Whisper Zoom” vs “Legacy Whisper”), and peptide input amounts into the simplePhos enrichment pipeline.

bioRxiv preprint doi: https://doi.org/10.1101/2025.10.08.681012; this version posted October 8, 2025. The copyright holder for this preprint (which was not certified by peer review) is the author/funder, who has granted bioRxiv a license to display the preprint in perpetuity. It is made available under aCC-BY 4.0 International license.

Supplementary Figure 3

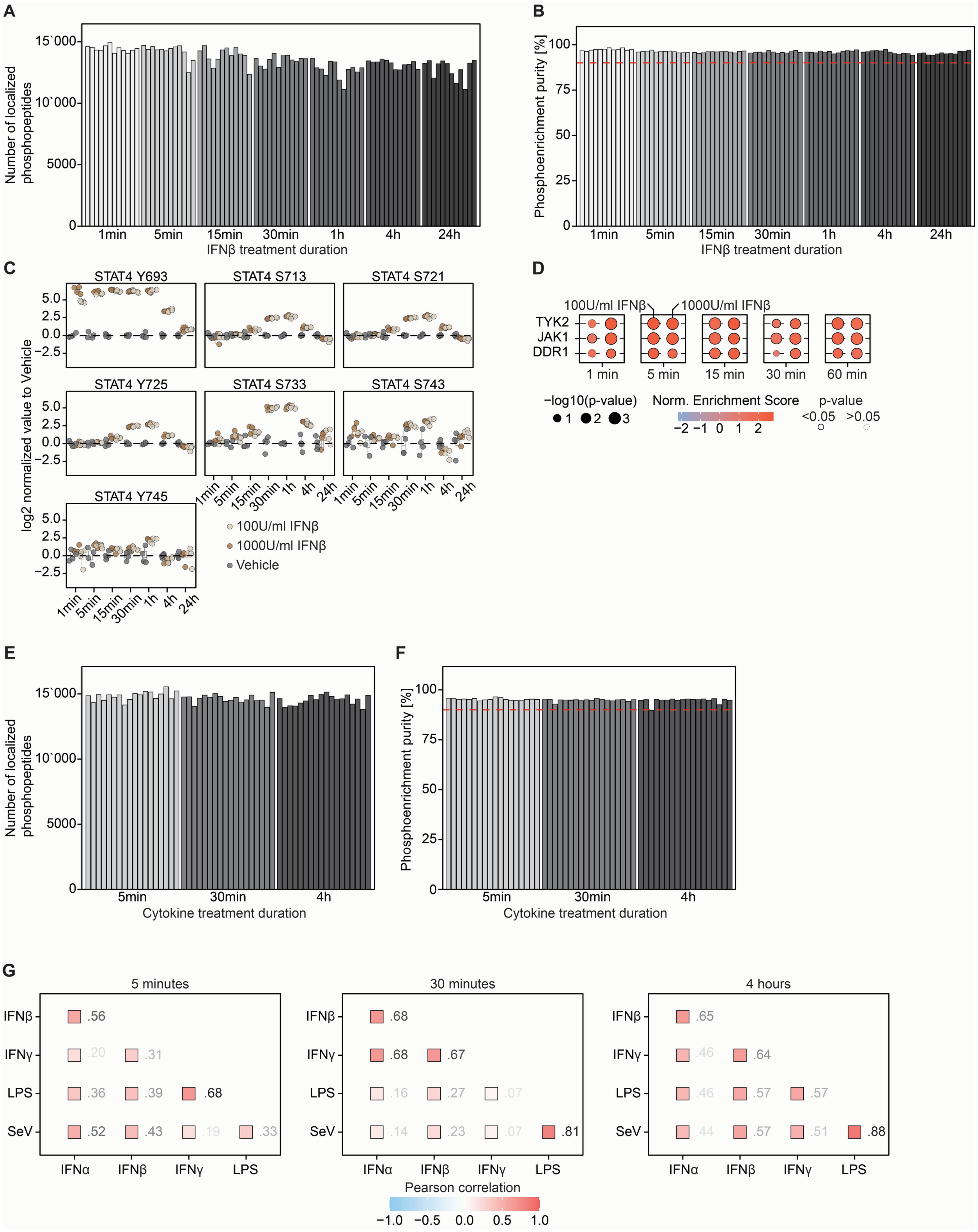

Supplementary Figure 3: *simplePhos* enables high-resolution temporal phosphoproteomic profiling of cytokine signaling in primary immune cells. (**A**) Number of localized phosphopeptides

bioRxiv preprint doi: https://doi.org/10.1101/2025.10.08.681012; this version posted October 8, 2025. The copyright holder for this preprint (which was not certified by peer review) is the author/funder, who has granted bioRxiv a license to display the preprint in perpetuity. It is made available under aCC-BY 4.0 International license.

in each CD4 T cell proteomics sample. (**B**) Purity of phosphopeptides across all samples; 90% threshold of phosphopeptide enrichment purity is shown in red. (**C**) Time-course boxplots showing phosphorylation kinetics of STAT4 phosphorylation sites. (**D**) Time-resolved kinase activity inference, depicting dynamic changes in activity of key kinases following interferon-β stimulation at two concentrations in CD4 T cells. (**E**) Number of localized phosphopeptides in each MDM proteomics sample. (**F**) Purity of phosphopeptides across all MDM samples; 90% threshold of phosphopeptide enrichment purity is shown in red. (**G**) Assessment of signaling similarity by Pearson correlation across phosphosites 5, 30 minutes, and 4 hours after cytokine stimulation of MDM samples.

bioRxiv preprint doi: https://doi.org/10.1101/2025.10.08.681012; this version posted October 8, 2025. The copyright holder for this preprint (which was not certified by peer review) is the author/funder, who has granted bioRxiv a license to display the preprint in perpetuity. It is made available under aCC-BY 4.0 International license.

Supplementary Figure 4

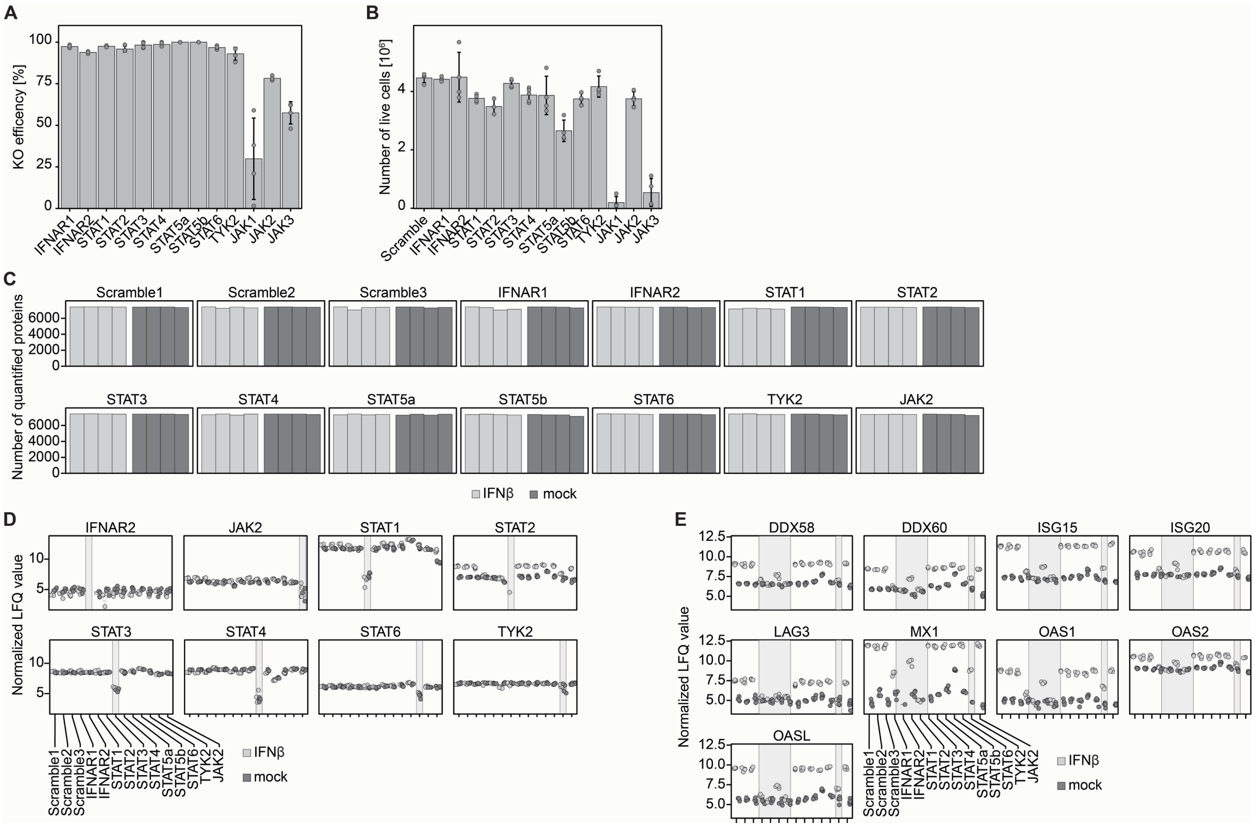

Supplementary Figure 4: CRISPR-mediated gene knockout in primary CD4 T cells coupled with global proteomics identifies key regulators of the type-I interferon response. (A) Editing efficiency of individual CRISPR knockouts in primary CD4 T cells. (**B**) Number of viable cells per million after genetic editing. **(C)** Number of quantified proteins detected in each knockout sample. **(D)** Boxplots showing normalized LFQ intensities of knockout target proteins across all knockout conditions. Conditions in which the target protein was disrupted are indicated with a grey background. **(E)** Boxplots of normalized LFQ intensities for selected canonical ISGs across knockout conditions. Knockouts with significant changes relative to scrambled controls are highlighted with a grey background.

bioRxiv preprint doi: https://doi.org/10.1101/2025.10.08.681012; this version posted October 8, 2025. The copyright holder for this preprint (which was not certified by peer review) is the author/funder, who has granted bioRxiv a license to display the preprint in perpetuity. It is made available under aCC-BY 4.0 International license.

Supplementary Figure 5

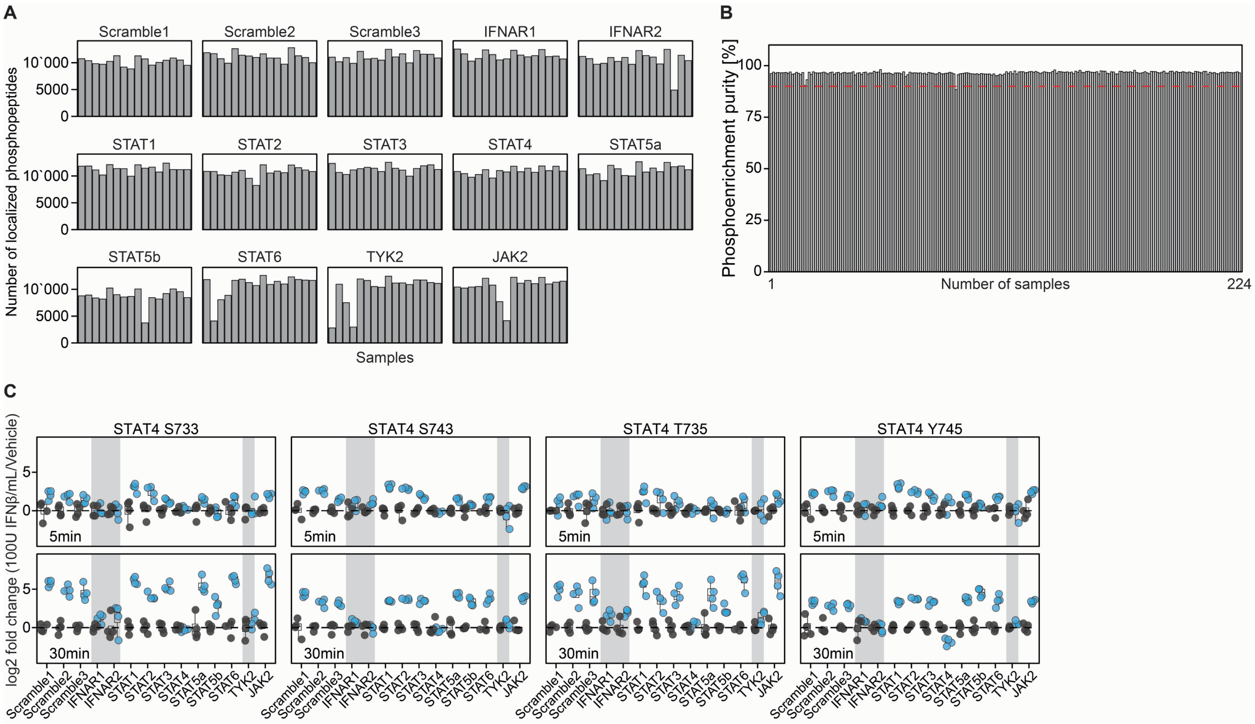

Supplementary Figure 5: *simplePhos* enables scalable phosphoproteomic profiling in saturating CRISPR knockout primary CD4 T cells. (**A**) Number of localized phosphopeptides in each proteomics sample. (**B**) Purity of phosphopeptides across all samples; 90% threshold of phosphopeptide enrichment purity is shown in red. (**C**) Quantitative analysis of phosphorylation sites on STAT4 in CRISPR knockout CD4 T cells following interferon-β stimulation for 5 and 30 minutes. Phosphorylation levels are shown relative to vehicle-treated controls with matching genetic backgrounds. Knockouts with significant changes relative to scrambled controls are highlighted with a grey background.

bioRxiv preprint doi: https://doi.org/10.1101/2025.10.08.681012; this version posted October 8, 2025. The copyright holder for this preprint (which was not certified by peer review) is the author/funder, who has granted bioRxiv a license to display the preprint in perpetuity. It is made available under aCC-BY 4.0 International license.

Supplementary Figure 6

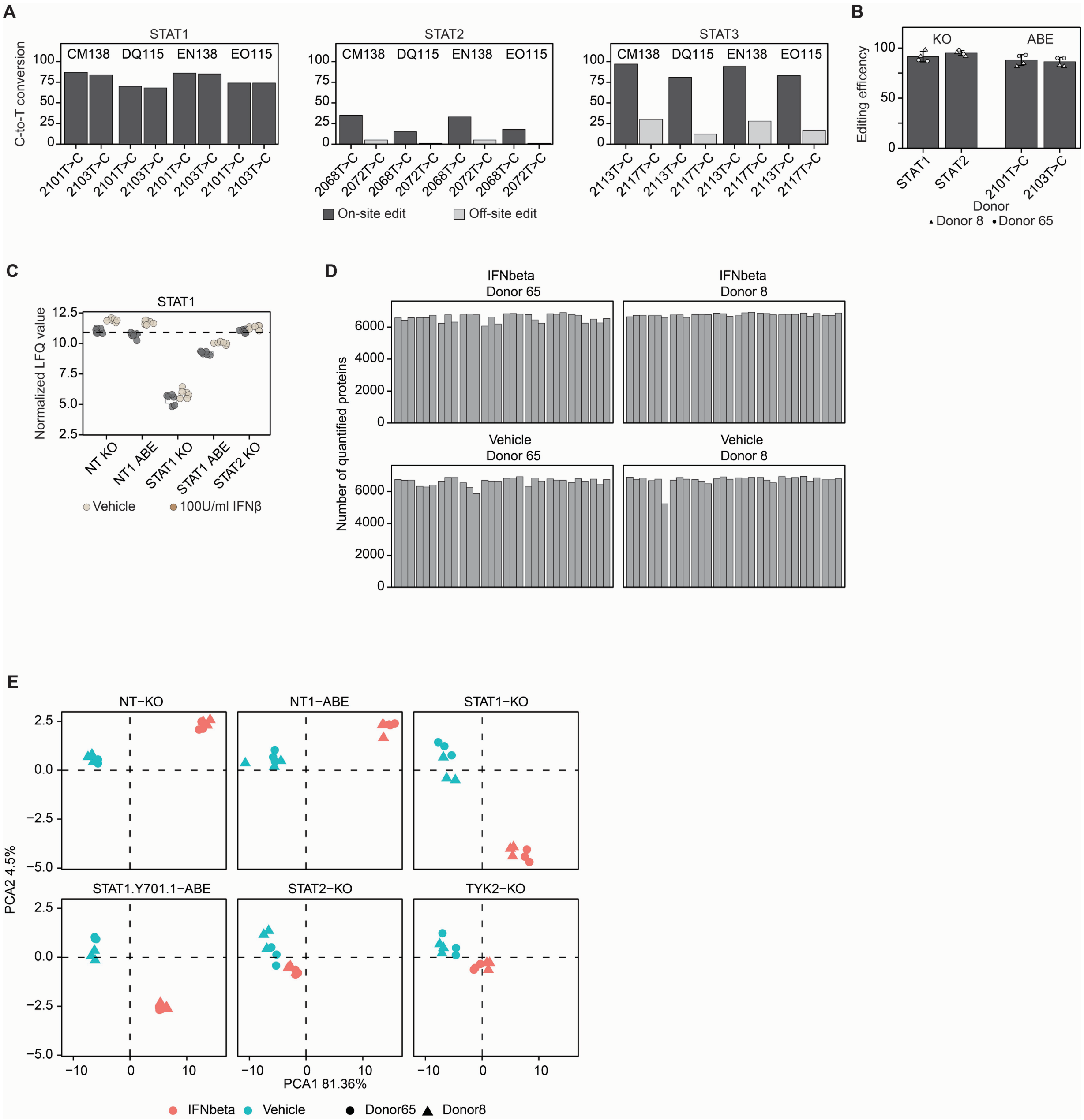

Supplementary Figure 6: Base editing in primary CD4 T cells enables precise interrogation of signaling events. (**A**) C-to-T conversion rates at different loci within STAT1, STAT2, and STAT3 under various electroporation programs (Lonza 4D Nucleofector). On-target edits are shown in dark, and off-target edits are shown in grey. (**B**) Editing efficiency of STAT1 and STAT2 knockout samples, as well as STAT1 Y701H base-edited samples, across two independent donors. (**C**) LFQ of STAT1 protein abundance across different genetic perturbations, knockout or base editing. (**D**) Number of quantified proteins in each proteomics sample. (**E**) Principal component analysis (PCA) based on significantly regulated proteins following IFN-β stimulation in the various genetically edited samples.
